# Preparation of superstructured comb polymers based on tadpole-shaped single-chain nanoparticles†

**DOI:** 10.1039/d4sc05650g

**Published:** 2024-10-03

**Authors:** Yangjing Chen, Zhiyu Hu, Zhigang Shen, Xiaoqiang Xue, Hongting Pu

**Affiliations:** a Department of Polymer Materials, School of Materials Science & Engineering, Tongji University Shanghai 201804 China puhongting@tongji.edu.cn; b Sinopec Shanghai Research Institute of Petrochemical Technology Co., LTD. Shanghai 201208 China; c Industrial College of Carbon Fiber and New Materials, School of Chemical Engineering and Materials, Changzhou Institute of Technology Changzhou Jiangsu 213000 China

## Abstract

Compared with the formation of individual elements, the creation of superstructures often yields exceptional properties. This approach has been applied to assemble diverse synthetic building blocks (molecules, macromolecules, inorganic nanoparticles, *etc.*) into highly organized constructs. In the present study, a novel comb polymer superstructure is developed *via* the grafting of tadpole-shaped single-chain nanoparticles (T-SCNPs) onto a high-molecular-weight linear backbone (H-LP). The resulting superstructure (comb of T-SCNPs), which utilizes T-SCNPs as building blocks, exhibits distinct rheological behavior in solution. The influences of the microstructure and related parameters (specifically the relaxation time (*τ*_R_) and mesh size (*ξ*) of the entangled chains) on the macroscopic properties (modulus and viscosity) of this complex topological structure in solution are investigated. Compared with conventional comb macromolecules (comb of F-LPs) and blends of SCNPs with high-molecular-weight polymers (SCNPs&H-LP), T-SCNP combs exhibit significantly reduced chain entanglement, faster *τ*_R_, and larger *ξ* in solution, resulting in a substantially decreased viscosity (up to 90%). Furthermore, our research underscores the intricate relationship between these rheological properties and the size and concentration of grafted T-SCNPs. As the size or concentration of T-SCNPs increases, the mesh size of the entangled chains expands, which leads to increased *τ*_R_ and decreased viscosity.

## Abstract 2

Super-structured comb polymers based on nanosized macro-monomers are prepared, which exhibit special rheological behavior due to their unique microstructure and related parameters (specifically mesh size and relaxation time).

## Graphical abstract

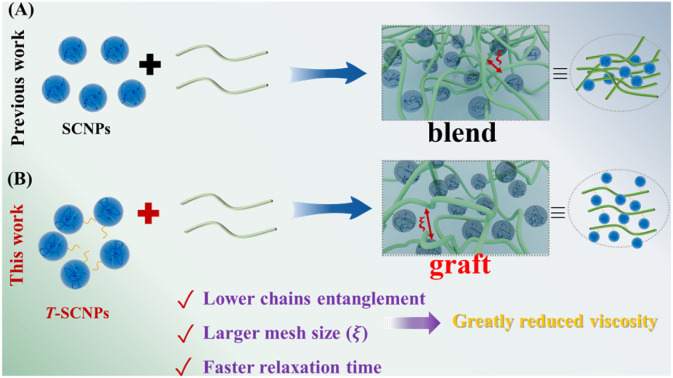

## Introduction

Complex polymer architectures, such as star-shaped,^1^ dendritic,^2^ grafted,^3^ bottle-brushed,^4^ or hyperbranched polymers,^5^ exhibit additional complexity that imparts unique physical properties. This inherent complexity and the resulting properties have facilitated the widespread application of complex polymer architectures in diverse fields, including drug delivery,^6^ bioimaging,^7^ catalysis,^8^ nanotemplates,^9,10^ photonics,^11^ and superelastomers.^12^ Therefore, polymer topology, including branching, cyclic, and cross-linked structures, is crucial in studying and designing the mechanical and rheological properties of bulk polymer materials.^13,14^ Among various topological structures, comb shaped polymers, which consist of a single backbone with multiple side chains, have recently garnered significant attention for their tailored and unique softening mechanical properties.^15–17^ These grafted polymers can encode mechanical properties through structural parameters such as backbone length, side chain length, and branch point spacing, thereby mimicking the mechanical characteristics of various soft materials.^18–21^ However, the fundamental chemical characteristics of these polymers, including intermolecular forces, conformational features, phase separation patterns, and crystalline structures, often hinder the ability to design their mechanical properties topologically.^22^ As a result, the task of understanding and engineering mechanical properties based on polymer topology remains a formidable challenge.^23^

In recent years, nanobuilding blocks (NBBs), which are composed of nanoparticle (NP) cores and precisely defined polymer chains, have emerged as innovative “colloidal molecules” for the bottom-up fabrication of functional materials and devices.^24^ These NBBs have shown promise in mechanically reinforcing polymer networks through the utilization of nanoparticles such as Fe^3+^,^25^ BaTiO^3+^,^26^ and metal–organic cages^27^ as cross-linking agents. Despite considerable^1–23^ advancements in this area, the design and synthesis of NBBs with precise structures, particularly those featuring specific NP core geometries and topologies, are still in the nascent stage, with only a few successful examples reported. Current synthetic methods of NBBs, such as selective surface modification,^28^ kinetically controlled synthesis,^29^ post-functionalization on cage-like inorganic compounds,^30^ and encapsulation,^31^ primarily involve complex synthesis processes, multiple preparation or purification stages, and insufficient control over the dimensions of both NP cores and polymer structures. A promising strategy for creating well-defined NBBs is the self-collapse of individual polymer chains through intramolecular cross-linking, as developed by Hawker's group.^32^ At very low concentrations, coil-to-globule transitions driven by various intramolecular reactions occur solely within individual polymer chains, resulting in what are known as single-chain nanoparticles (SCNPs).^33–38^ SCNPs, characterized by their ultrafine sizes and “secondary” structures, have attracted considerable interest due to their applications in sensing,^39^ catalysis,^40,41^ bioimaging,^42^ and drug delivery.^43^

Furthermore, Hawker *et al.* demonstrated that the single chain polymer chemistry could be extended to linear block copolymers where the coil-to-globule transition only occurred within one block containing cross-linkable groups. This gave rise to the formation of a tadpole-shaped NBB (T-SCNP) where the crosslinked block formed the “head”, attached by an un-cross-linked polymer “tail”.^44^ This provides the possibility of preparing more complex topological structures using T-SCNPs, as the cross-linked “tail” can be functionalized and grafted into the linear polymer skeleton through grafting methods, forming a novel comb shaped polymer. Interestingly, when internal crosslinking of T-SCNPs occurs *via* dynamic covalent bonds, this unique comb-shaped polymer can reversibly transform into traditional comb-shaped polymers. This process offers a platform for developing novel topology-reversible transition materials.

Moreover, using T-SCNPs as grafting units allows for orthogonal control over the interaction of the “heads” with the surface and the properties of the comb. Consequently, this method can achieve tasks that existing methods cannot.^45^ Compared with traditional comb polymers, comb polymers with special topological structures prepared by grafting T-SCNPs with specific sizes and shapes as grafting units can significantly alter the chain entanglement behavior of the system and the relaxation process of chain segments due to the size effect of their side chains. This, in turn, affects their macroscopic properties, especially the change in rheological behavior. To the best of our knowledge, there has been limited exploration into how the topological structure and size of the side chains attached to comb polymers affect their microstructure, including entanglement behavior of chain segments, relaxation rate, and mesh size of the entangled chain. The topic of chain entanglement has been of interest to polymer scientists for decades, as it has significant implications for adjusting macroscopic properties through the microstructure of the system.^46–48^

## Results and discussion

**Scheme 1:**
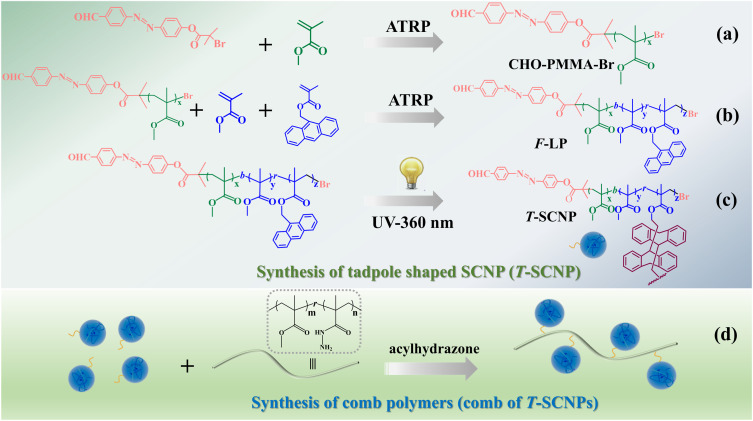
Synthesis route of the polymers: (a) macromolecular initiator (CHO–PMMA–Br). (b) Linear precursor of T-SCNP (F-LP). (c) Tadpole shaped single-chain nanoparticle (T-SCNP). (d) Comb polymer (comb of T-SCNPs).

The synthetic method utilized in this study is depicted in Scheme 1. A random copolymer (F-LP) containing methyl methacrylate (MMA), anthracenyl methacrylate (AMA), and an aldehyde end group was synthesized *via* atom transfer radical polymerization (ATRP), employing CHO–PMMA–Br as a macroinitiator (Scheme 1a and b). The successful preparation of CHO–PMMA–Br was verified using gel permeation chromatography (GPC), proton nuclear magnetic resonance (^1^H NMR), and mass spectrometry (MALDI-TOF MS). The SEC curve of the polymer (CHO–PMMA–Br) exhibited a single peak with a small dispersity index (*Đ*) (Fig. S3 in the ESI†), which is characteristic of ATRP. In the ^1^H NMR spectrum (Fig. S4 in the ESI†), proton peaks corresponding to the polymer were evident, and the number-average molecular weight (*M*_n_) calculated from NMR data was 2700 g mol^−1^, which is consistent with the GPC result (3200 g mol^−1^). The mass spectrum of CHO–PMMA–Br showed a Gaussian distribution (Fig. S5 in the ESI†), with the main peak corresponding to a molecule where the bromine end group was replaced by a sodium ion and adjacent major peaks differing by 100.1, corresponding to the MMA monomeric unit. Minor peaks represented molecules with a potassium ion at the chain end, with adjacent minor peaks also differing by 100.1. The collapse process of the liner precursor was conducted using a copolymer (F-LP) through the photodimerization of pendant anthracene groups at a low polymer concentration (*c* = 1 mg mL^−1^) to produce tadpole-shaped single-chain nanoparticles (T-SCNPs). In this process, functional copolymers were excited, leading to internal dimerization of the highly aromatic pendant groups under an inert atmosphere (Scheme 1c). The composition of the F-LP was ascertained using NMR calculations, which revealed the ratio of the methylene protons of methyl methacrylate units at 3.51 ppm and the overall aromatic protons of the anthracene segments ranging from 7.51–8.52 ppm, confirming that the copolymer (F-LP) comprised 10 mol% of the anthracene segments. Following dimerization, the signals for the aromatic protons of the anthracene segments shifted from 7.51–8.52 ppm to 6.89 ppm, as shown by ^1^H NMR analysis (Fig. S6 in the ESI†).

Table S1 (in the ESI†) lists the molecular weights (*M*_n_ and *M*_w_), dispersity index (*Đ*), sizes (*R*_h_), and glass transition temperatures (*T*_g_) of both F-LP and T-SCNP. The intramolecular anthracene dimerization process resulted in a decrease in the hydrodynamic radius (*R*_h_) of the resulting T-SCNPs, leading to a noticeable reduction in *M*_n_ due to a transition from a coil to a globe structure. Additionally, the SEC trace of the T-SCNPs indicated a longer retention time than that of its linear precursor after the photoinduced internal crosslinking process (Fig. S7 in the ESI†). The UV-Vis spectra of F-LP exhibit a characteristic absorption peak for anthracene moieties between 325 and 400 nm. Following irradiation, the intensity of the absorption peak decreased indicating the dimerization of pendant anthracene units, as depicted in Fig. S8 (in the ESI†). The glass transition temperature (*T*_g_) is a critical property influencing the processing performance of polymers. It represents the temperature at which amorphous polymers transition from a glassy state to a highly elastic state. The movement of chain segments in T-SCNPs is restricted, leading to reduced degrees of freedom of the chains. Fig. S9 (in the ESI†) compares the *T*_g_ values of T-SCNPs and F-LP. Compared with that of F-LP (*T*_g_ 122 °C), the *T*_g_ of T-SCNPs was found to be 132 °C, indicating that the internal crosslinking structure of SCNPs restricts chain segment mobility.

The tadpole-shaped SCNP (T-SCNP), featuring a segment with an aldehyde functional group at the terminal, is capable of reacting with high-*M*_w_ linear polymers (H-LPs) that contain hydrazide side groups, facilitating the grafting of T-SCNPs onto the linear backbone. The structure of the H-LP was characterized using ^1^H NMR and GPC, as indicated in Fig. S10 and S11 in the ESI.† The SEC curve exhibited a single peak with a *Đ* of 1.92 and *M*_w_ of 10.8 kDa, which is consistent with the characteristics of conventional free radical polymerization. The positions of each peak in the NMR spectrum correspond to the protons of the polymer.

Additionally, comprehensive characterization *via*^1^H NMR, DLS, SEC, SAXS, and diffusion-ordered NMR spectroscopy (DOSY NMR) provided substantial evidence for the successful synthesis of a superstructure comb polymer (comb of T-SCNPs) using T-SCNPs as graft units. The complete reaction between the aldehyde and hydrazide groups was confirmed by the disappearance of the aldehyde proton peak and the appearance of new peaks representing hydrazone bonds in the NMR spectrum (Fig. S12 in the ESI†). The NMR results revealed that the positions of the two spectral lines were almost identical, with minor variations in the relative intensities of the peaks. With the formation of the comb of T-SCNPs, the disappearance of peak a (10.08 ppm), which corresponds to the hydrogen peak of the –CHO– group of F-SCNPs, was noted. Moreover, new peaks b′ and c′ in the spectrum of the comb of T-SCNPs were identified, corresponding to the protons of the –CH=NH– (10.72 ppm) and NH (8.31 ppm) groups, respectively.

**Fig. 1:**
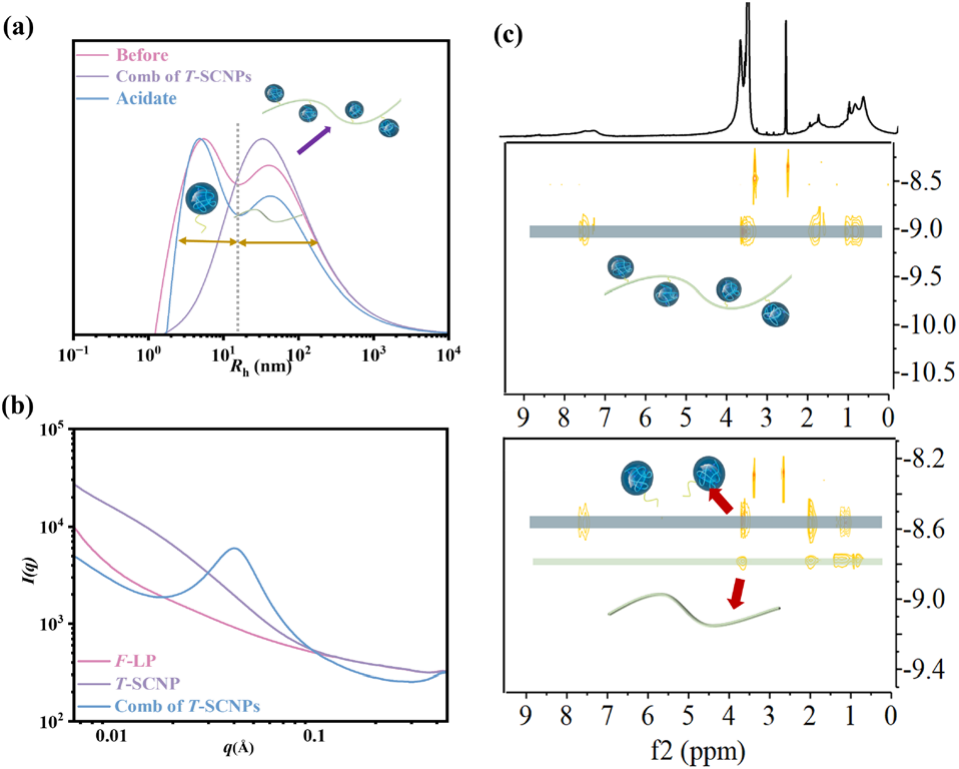
Chemical structure of the comb of T-SCNPs and its precursor. (a) Hydrodynamic radius distribution *f*(*R*_h_). (b) 1D SAXS profiles. (c) DOSY-NMR spectra of the comb of T-SCNPs (top) and its precursor (bottom).

The SEC curves before and after the grafting reaction indicated a transformation from two distinct peaks, corresponding to the T-SCNPs and the high-*M*_w_ linear polymer (H-LP), to a single peak, indicating the transition from two separate components to one unified component (Fig. S13 in the ESI†). However, a notably higher *Đ* was observed, potentially due to variations in the grafting efficiency across different chains, resulting in diverse hydrodynamic volumes in solution. The dynamic light scattering (DLS) results support the SEC data, showing a transition from two peaks to a single peak with an expanded size distribution after the reaction (Fig. 1a). Remarkably, the acylhydrazone bond serves as a dynamic covalent bond. Upon the addition of a small amount of acid, coupled with a system pH of about 6.5, the acylhydrazone bond is cleaved, resulting in the detachment of T-SCNPs from the H-LP. This dissociation is evidenced by a return from a single peak to the original two peaks in the DLS results.

Fig. 1b displays the one-dimensional (1D) SAXS intensity results for the F-LP, T-SCNPs, and comb of T-SCNPs. The SAXS experiment was performed at the BL16B beamline of the Shanghai Synchrotron Radiation Facility (China). Initially, the results indicate a qualitative difference between linear polymers and SCNPs. The extended polymer precursor exhibited a region of linear decrease at low *q* values, whereas the compact SCNP presented higher order structures. This phenomenon is described by the relationship *I*(*q*) ∝ *q*^*p*^, where *p* is the Porod exponent. A Porod exponent of 4 indicates an ideal sphere, a range of 3 < *p* < 4 suggests a flexible protein, a value of 2 corresponds to an unfolded, random chain, and *p* = 1 signifies a fully extended chain.^49^ For the comb of T-SCNPs, the 1D SAXS curve displays a distinct peak at 0.47 nm, reflecting the locally ordered structure of the T-SCNPs. Additionally, the distance between the T-SCNP domains within the comb of the T-SCNPs was precisely determined to be 13.36 nm, as calculated using Bragg's equation (*d* = 2π/*q*).

Finally, the effectiveness of grafting T-SCNPs onto the H-LP was evaluated using DOSY NMR, a technique that exploits the exponential decay of signals due to molecular self-diffusion. DOSY NMR provides a two-dimensional analysis where the first dimension (*F*_2_) reflects conventional chemical shifts and the second dimension (*F*_1_) corresponds to the self-diffusion coefficient (*D*).^50^ This method is highly sensitive and adept at detecting even trace amounts in complex mixtures. For this study, DOSY NMR of the resulting comb of T-SCNPs and its precursors was conducted in dilute DMSO. As depicted in the DOSY map (Fig. 1c), distinct diffusion coefficients are observed in the SCNP&H-LP mixture, indicating separate compositions of T-SCNP and H-LP. The uniform diffusion coefficients across all the protons within the comb of T-SCNPs confirmed the efficient grafting of T-SCNPs onto the H-LP.

The inclusion of nanoparticles, whether inorganic,^51^ grafted with SiO_2_,^52^ or dendrimers,^53^ alters the rheological properties of polymers, causing a significant decrease in system viscosity. Several theories, such as the free volume effect, nanoconfinement effect, nanoparticle surface slip effect, shear banding effect, and shear thinning effect induced by nanoparticles, have been proposed to explain this change. However, Goldansaz and colleagues,^54^ in their study of dendritic polyethylene/polystyrene composites, concluded that these effects alone cannot fully explain the viscosity reduction observed. This underscores the complexity arising from interactions between nanoparticles and polymer chains in heterogeneous composite systems, further compounded by chemical compositional differences. Therefore, developing broadly applicable theoretical models across different systems remains challenging. In nanoparticle–polymer composites, both the kinetic properties of the polymer chains and the dynamic behaviors of the nanoparticles play critical roles. The diffusion kinetics of nanoparticles are closely linked to their size relative to the characteristic length of polymer chains, with different types of nanoparticles exhibiting distinct diffusion mechanisms within the polymer melt.

Mackay *et al.*^55^ incorporated SCNPs of polystyrene into a polystyrene melt, resulting in an 80% viscosity reduction with just a 1% volume of SCNPs, despite their chemical similarity to the polymer chains. This phenomenon, which occurs without specific enthalpic interactions between the nanoparticles and melt chains, remains inadequately explained by current theories. Recent molecular dynamics simulations by Qian and colleagues indicate that the reduced internal degrees of freedom within SCNPs lead to decreased friction on the surrounding molten polymer chains.^56^ This insight illustrates the mechanism responsible for viscosity reduction in polymer systems due to SCNPs.

**Fig. 2:**
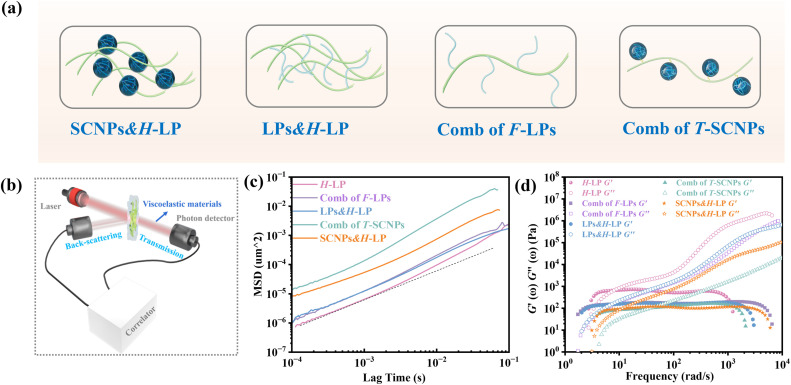
(a) Scheme of different systems. (b) Transmission and backscattering modes in a basic DWS experiment. (c) Mean square displacement, Δ*r*^2^(*τ*), using TiO_2_ particles (*R*_h_ = 180 nm) embedded in different systems. (d) Frequency spectra of *G*′ and *G′′* of different systems.

We investigated the direct grafting of T-SCNPs onto linear main chains *via* an aldehyde–acylhydrazone reaction, contrasting this with the simple addition of SCNPs to polymer melts or solutions in composite form. Our focus was on exploring the potential performance of novel comb topological polymers in solution. For comparison, mixtures of SCNPs with high *M*_w_ linear matrix polymers (SCNPs&H-LP) and traditional comb polymers (comb of F-LPs) were prepared, where T-SCNP precursor polymers were grafted onto the main chains of the H-LP. Additionally, mixtures of SCNP precursor polymers with the H-LP (LPs&H-LP) were prepared (Fig. 2a). The amount of grafted F-LP corresponded to the molar quantity of T-SCNPs in the comb of the T-SCNP system, which was consistent with the molar quantities of SCNPs and LPs added to the two composite systems (SCNPs&H-LP and LPs&H-LP). The structural details of the LPs, SCNPs, and comb of F-LPs are provided in the ESI, as illustrated in Fig. S14–S16 in the ESI.†

Microrheological experiments were conducted to explore the varied rheological behaviors exhibited by these systems in solution, aiming to further understand chain entanglement, restricted chain mobility, and disentanglement effects induced by SCNPs grafted onto high *M*_w_ linear polymers. A contemporary microrheology technique involves tracking the movement of a small particle probe, typically a colloidal microsphere, to minimize mechanical disturbance of the medium (Fig. 2b).^57^ Colloidal particles are ideal probes because they induce minimal perturbations in the structure and dynamics of soft matter at thermal energies of about *k*_B_T (where *k*_B_ is Boltzmann's constant and *T* is the absolute temperature). This characteristic enables precise measurement of the rheological properties of materials at scales ranging from micrometers to submicrometers. In contrast to conventional rotational mechanical rheometers, microrheology studies impose virtually negligible strain on the material during measurement.^52^

Fig. S17 in the ESI† illustrates the ICF curves of various systems, showing that the interchain friction coefficient in each system decays to 0 over time, indicating complete chain relaxation. The H-LP system demonstrated prolonged relaxation due to extensive interchain entanglement; however, the addition of SCNPs to the system significantly decreased the chains' relaxation rate, consistent with previous findings.^56^ Interestingly, the comb of the T-SCNP system exhibited a notably faster relaxation rate compared to the SCNPs&H-LP system. We hypothesize that grafting SCNPs into the H-LP not only reduces friction between linear chains through steric effects but also reduces chain density around SCNPs, thereby increasing system free volume. The comb of F-LPs and LPs&H-LP systems show minimal differences; however, compared with that of the H-LP system, the relaxation rate of entangled chains in the two systems is enhanced, possibly because of the plasticizing effect of the low *M*_w_ linear polymers.

To characterize the translational dynamics of polymer chains, we calculated the MSD of tracer particles in polymer solutions across different systems on the basis of the velocity autocorrelation function for a Brownian particle (eqn (1) and (2))1
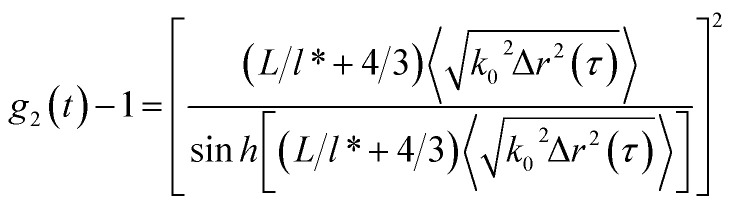
where *L* is the thickness of the sample and l* is the sample transport mean free path of the scattered light. *k*_0_ = 2π/*λ*, where *λ* is the laser wavelength.

2 〈Δ*r*^2^(*τ*)〉 = *δ*^2^(1 − e^(−*τ*/*τ*_c_)*a*^)

where *δ*^2^ represents the amplitude of particle motion, *a* is the short-time logarithmic slope, *τ* is the lag time, and *τ*_c_ is the characteristic relaxation time of the particle in the polymer solutions.

Fig. 2c displays MSD curves of tracer particles (TiO_2_, 360 nm) in the polymer solutions of various systems. Initially, at short lag times, the MSDs exhibit a linear trend proportional to the lag time (Δ*r*^2^(*τ*)–*τ*), indicating pure diffusive motion of the tracer particles and a microenvironment resembling a viscous liquid. In the entangled regime (at intermediate lag times), MSDs display a power-law behavior with lag time, with a slope slightly less than one (Δ*r*^2^(*τ*)–*τ*^0.85^), indicative of polymer entangled network cage formation characteristic of viscoelastic behavior. The tracer particles in the comb of the T-SCNP system exhibit much higher MSD values than those in other systems, corresponding to the highest diffusion coefficient. The Laplace transform of the MSD corresponds to the complex shear modulus (G*), which is evaluated using the generalized Stokes–Einstein equation (eqn (3)).^58^ This connection facilitates the computation of the storage modulus (*G*′) and loss modulus (*G′′*) using eqn (4).3
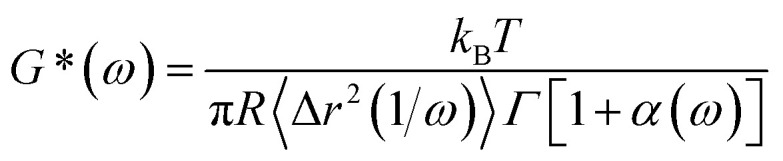
where *R* is the diameter of the tracer particles.4
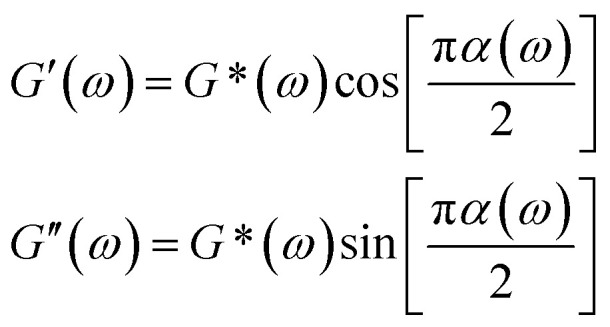
where *τ* = 1/*ω*, *α*(*τ*) = ∂[ln〈Δ*r*^2^(*τ*)〉]/∂[ln *τ*] and *Γ* is the gamma function.

Fig. 2d depicts the frequency-dependent modulus variation across several systems. This demonstrates that the reduced chain entanglement within the system leads to decreased friction during the disentanglement process. Consequently, the comb of the T-SCNP system has the lowest *G*′′, which is consistent with MSD findings across different systems.

**Fig. 3:**
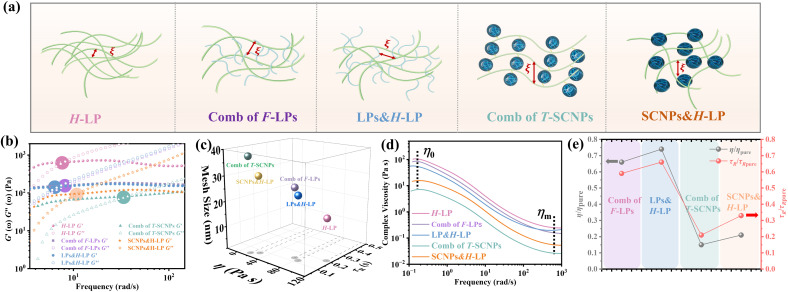
(a) Scheme of mesh size (*ξ*) of entangled chains in different systems. (b) The relaxation time (*τ*_R_) of chains in different systems. (c) The mesh size (*ξ*) of entangled chains in different systems. (d) Complex viscosity curves of polymers in different systems. (e) The relative viscosity *η*/*η*_pure_ and *τ*_R_/*τ*_R pure_ for the different systems; *η* and *τ*_R_ are the viscosities and relaxation times of the comb of F-LPs, LPs&H-LP, comb of T-SCNPs, and SCNPs&H-LP respectively; *η*_pure_ and *τ*_R pure_ are the viscosity and relaxation time of the linear matrix polymer (H-LP).

Therefore, we believe that integrating SCNPs into a linear polymer matrix through grafting induces substantial alterations in the system's microstructure, resulting in discernible variations in the macroscopic properties. The macroscopic behavior of polymers is intricately linked to their microstructural configuration. The unique rheological characteristics observed in the T-SCNP comb system in solution stem from an enlarged mesh size (*ξ*) between the interlocked chains. As depicted in Fig. 3a, grafting T-SCNPs onto high *M*_w_ linear main chains is believed to increase the distance between these chains, thereby reducing chain entanglement. Similarly, incorporating mixed-form SCNPs into high *M*_w_ linear resins is anticipated to yield a comparable effect. However, SCNPs feature internal loops and clusters of small loops referred to as “domains,” which are essential for filling space at higher polymer concentrations. These entanglements are thought to be minimized within the T-SCNP comb structure. In contrast, in the comb of F-LPs and LPs&H-LP systems, intermolecular entanglements persist between low *M*_w_ linear polymers and high molecular weight matrix resins, limiting effective reduction of chain entanglement.

In DWS, the plateau modulus *G*_0_ is related to the diffusivity of tracer particles through the equation *G*_0_ = *k*_B_T/(6π*αδ*^2^). Additionally, the mesh size *ξ*, which denotes the spacing between entangled polymer chains, can be determined using eqn (5).^59^

5 *ξ* = (*k*_B_*T*/*G*_0_)^1/3^

The introduction of SCNPs into the H-LP system (SCNPs&H-LP) led to a significant increase in *ξ*, from 17.32 to 31.07 nm (as indicated in Table 1), indicating that SCNPs increase the free volume within the system. In the comb of the T-SCNP system, *ξ* further increases to 35.83 nm compared with that of the SCNPs&H-LP system. Moreover, higher chain entanglement within the system hinders chain segment relaxation due to intermolecular friction, resulting in longer relaxation times. A crossover of the *G*′ and *G*′′ is observed along with the determination of the terminal relaxation time (*τ*_R_), which is the reciprocal of the crossover frequency.^60^ For long linear chains, *G*′ and *G*′′ curves typically intersect at low frequencies, reflecting chain disentanglement. Comparing relaxation times (*τ*_R_) of H-LP, LPs&H-LP, and the comb of F-LP systems, *τ*_R_ decreases from 0.2 s to 0.13 s and 0.12 s for LPs&H-LP and the comb of F-LP systems, respectively, as shown in Fig. 3b. However, there is a minimal difference in *τ*_R_ between LPs&H-LP and F-LP systems. Notably, the *τ*_R_ of the T-SCNP comb system is significantly reduced, showing a fivefold decrease compared with that of the H-LP and a twofold decrease compared with that of SCNPs&H-LP, as detailed in Fig. 3c and Table 1. Thus, the impact of SCNPs on viscosity primarily arises from its size effect, which increases the free volume around linear resins, thereby reducing the friction coefficient.

**Table 1:** The microrheological characteristics of different systems

Samples	6*δ*^2^ × 10^5^^a^ (μm^2^)	*η*_0_ a (Pa s)	*η*_m_ × 10^2^^a^ (Pa s)	*G*_0_ a (Pa)	*τ*_R_ × 10^2^^b^ (s)	Mesh size^c^ (nm)
H-LP	1.05	111.8	29.2	780	20.3	17.32
Comb of F-LPs	5.46	73.8	12.9	185	12.1	27.98
LP&H-LP	7.29	83.1	19.8	238	13.5	25.71
Comb of T-SCNPs	16.32	16.7	5.8	88	4.3	35.83
SCNPs&H-LP	12.12	27.9	8.9	135	7.2	31.07

a Data acquired from DWS.

b Data acquired from the crossover of *G*′ and *G*′′.

c Data acquired from the corresponding theoretical calculation using eqn (5).

The viscosity curves (Fig. 3d) demonstrate that the comb of the T-SCNP system has the lowest zero-shear viscosity (*η*_0_). As the frequency increases, chains begin to disentangle, leading to the viscosity reduction characteristic of a Newtonian fluid. At higher frequencies, where chain entanglements are fully resolved, viscosity stabilizes. In this regime, viscosity is solely influenced by the intrinsic structure of polymers, as entangled chains are fully released from interaction. The viscosity curve indicates that *η*_m_ of the T-SCNP comb is one-fifth that of the H-LP polymer, highlighting the significant impact of T-SCNPs on the polymer conformation in solution. Fig. 3e presents the ratios of zero-shear viscosities (*η*/*η*_pure_) and relaxation times (*τ*_R_/*τ*_R pure_) of various systems compared to their respective matrix polymer chains. For the comb of F-LPs and F-LPs&H-LP systems, the viscosity is moderately reduced with ratios of *η*/*η*_*p*ure_ = 0.66 and 0.75, respectively. In contrast, the viscosity ratio for the SCNP&H-LP system is significantly reduced to 0.25. This ratio can be further decreased to 0.15 with the comb of the T-SCNP system. These findings align well with the reduction in relaxation time, where the *τ*_R_/*τ*_R pure_ of the T-SCNP comb system is 0.21, indicating a substantial increase in the chain segment disentanglement rate. This accelerated chain dynamics is likely attributable to the disentanglement effect induced by T-SCNPs on the side chains.

To test our hypothesis that the size effect of T-SCNPs introduces additional free volume, accelerating the disentanglement of polymer chains and reducing viscosity, we synthesized T-SCNPs of varying sizes by adjusting the crosslinking group content in linear precursors (F-LPs) and grafting them onto high *M*_w_ linear polymers. Fig. S18 and S19,† along with Table S2 (in the ESI†), detail GPC and DLS data for three T-SCNPs of different sizes, demonstrating that increased crosslinking led to more compact T-SCNPs with smaller *R*_h_ and, consequently, lower *M*_n_ (Table S2 in the ESI†). The UV spectra of these T-SCNPs showed that a relatively high anthracene content increased the absorption peak intensity at 360–400 nm (Fig. S20 in the ESI†). However, upon nanoparticle formation, the absorption peak intensity decreased due to the combination of pendant anthracene units after irradiation. ^1^H NMR analysis of F-LPs with varying anthracene contents and T-SCNPs of different sizes indicated that higher anthracene content increased the proton peak intensity at 7.21–8.52 ppm. The dimerization of anthracene caused a shift in the peak toward 6.53 ppm, suggesting extensive involvement of anthracene rings in the dimerization reaction (Fig. S21 in the ESI†).

Finally, we evaluated the folding of linear polymers containing different AMA groups using characteristic viscosity, the Zimm branching factor *g*′, and the Stokes ratio *R*_g_/*R*_h_. Increasing the content of the linear polymer AMA led to enhanced internal crosslinking, enabling SCNPs to adopt a more spherical three-dimensional structure and reducing interchain entanglement. T-SCNP3, which has the highest degree of internal crosslinking, presented lower viscosity and *g*′ values than T-SCNP1, T-SCNP2, and their linear precursors (Fig. S22 and S23 in the ESI†). Additionally, *R*_g_/*R*_h_ is critical for describing molecular configuration in solution. A lower *R*_g_/*R*_h_ value indicates a more compact structure in solution.^61^ As shown in Fig. S24,†*R*_g_/*R*_h_ values for F-LP1-3 ranged from 1.2–1.4, whereas those for T-SCNP1-3 varied from 0.5 to 0.8, demonstrating that SCNPs adopt a more compact structure in solution than their linear analogs. Notably, T-SCNP-3, with the highest degree of internal crosslinking, exhibited the lowest *R*_g_/*R*_h_ value, highlighting its exceptionally compact molecular configuration.

**Fig. 4:**
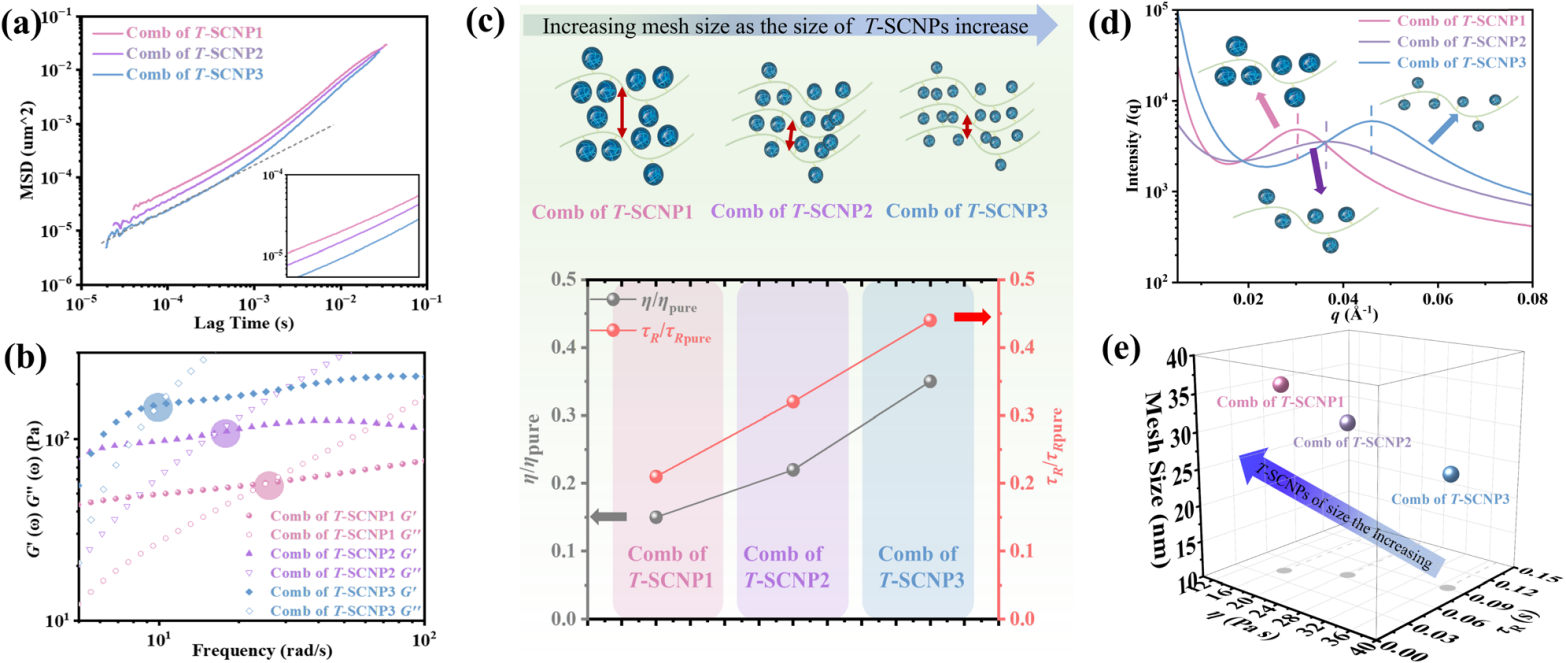
(a) MSD curves of combs of T-SCNP systems with different sizes of T-SCNPs. (b) The relaxation time (*τ*_R_) of combs of T-SCNPs with different sizes of T-SCNPs. (c) The relative viscosity *η*/*η*_pure_ and *τ*_R_/*τ*_R pure_ for the different systems; *η* and *τ*_R_ are the viscosity and relaxation time of the comb of T-SCNP1-3 respectively; *η*_pure_ and *τ*_R pure_ are the viscosity and relaxation time of the linear matrix polymer (H-LP), respectively. (d) 1D SAXS profiles of combs of T-SCNPs with different sizes of T-SCNPs. (e) Mesh size of combs of T-SCNPs with different sizes of T-SCNPs.

According to microrheological studies, enlarging the size of T-SCNPs leads to an increase in the MSD of tracer particles across three different systems (Fig. 4a). For example, the MSD for the comb of T-SCNP1 is 16.32 × 10^−5^ μm^2^, almost threefold greater than that for the comb of T-SCNP3, which is 6.39 × 10^−5^ μm^2^. Additionally, the *τ*_R_ values vary significantly across these systems, with Fig. 4b displaying *τ*_R_ values for each system. The *τ*_R_ for the comb of T-SCNP1 is 0.043 s, considerably faster than that for the comb of T-SCNP2 (0.065 s) and T-SCNP3 (0.089 s). The viscosity ratio for the comb of T-SCNP1, *τ*_R_/*τ*_R pure_, is 0.21, whereas it increases to 0.32 for the comb of T-SCNP2 and further to 0.44 with the smaller T-SCNP3. These findings align well with the viscosity data (Fig. 4c).

It is hypothesized that maintaining the same SCNP concentration with increased SCNP volume necessitates greater distances between polymer chains, thereby reducing the local chain concentration and accelerating the chain relaxation process. The low-*q* SAXS peak measurements reveal *d*-spacings for the comb of T-SCNP1, T-SCNP2, and T-SCNP3 at 21.5 nm, 17.3 nm, and 13.9 nm, respectively, indicating that larger SCNPs must be spaced further apart to maintain a constant T-SCNP concentration (Fig. 4d). This is supported by *G*_0_ calculations of polymer entanglement mesh size, with the *ξ* for the comb of T-SCNP1 being 35.83 nm, larger than those for the comb of T-SCNP2 (31.15 nm) and T-SCNP3 (25.52 nm) systems. Fig. 4e and S25 (in the ESI†) indicate the lowest viscosity in the comb of T-SCNP1, which is consistent with fewer intermolecular entanglements.

**Fig. 5:**
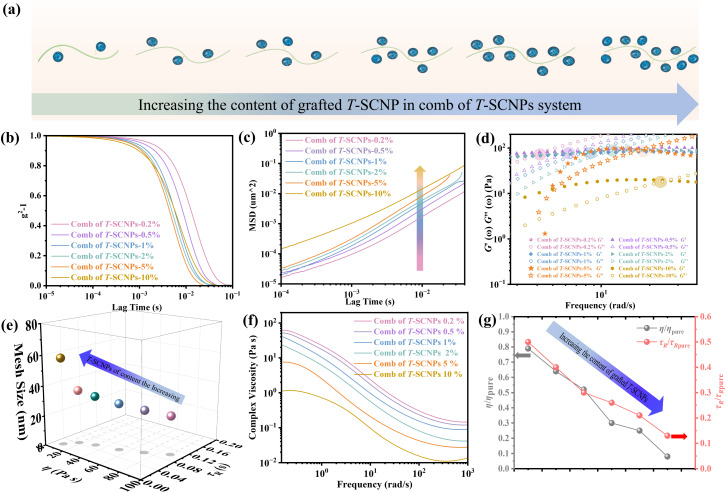
(a) Scheme of the comb of T-SCNPs with different contents of T-SCNPs. (b) ICF curves of the combs of T-SCNPs with different contents of T-SCNPs. (c) MSD curves of combs of T-SCNPs with different contents of T-SCNPs. (d) Relaxation time (*τ*_R_) of combs of T-SCNPs with different contents of T-SCNPs. (e) Mesh size of combs of T-SCNPs with different contents of T-SCNPs. (f) Complex viscosity curves of combs of T-SCNPs with different contents of T-SCNPs. (g) The relative viscosity *η*/*η*_pure_ and *τ*_R_/*τ*_R pure_ for the different systems.

Previous studies have shown that adding SCNPs reduces the viscosity of the polymer solution by increasing the free volume at the polymer/SCNP boundary. In contrast, past reports have documented that incorporating inorganic nanoparticles into polymer melts increases the system's viscosity once the nanoparticle loading exceeds a certain threshold, leading to the formation of NP aggregates that significantly impede melt chain relaxation.^62^ Here, the rheological properties of combs of T-SCNPs with different grafting densities were examined, as shown in Fig. 5a. It is believed that increasing the grafting density of T-SCNPs reduces the entanglement density among polymer chains due to steric hindrance. The ICF curves in Fig. 5b demonstrate that higher grafting densities accelerate the relaxation rate of the system. Concurrently, the most intense Brownian motion within the system correlates with higher MSD values (Fig. 5c). The modulus *versus* the frequency curve (Fig. S26 in the ESI†) shows a marked decrease in *G*′′ with increasing T-SCNP grafting content, with the intersection point of the two moduli moving toward higher frequencies, indicating quicker *τ*_R_ (Fig. 5d). As indicated in Fig. 5e and Table S3 (in the ESI†), the *G*_0_ of the system decreases as T-SCNP grafting content increases. At a 10% grafting rate, the *ξ* significantly increased from 17.32 nm for the pure H-LP to 57.82 nm. This finding suggests that the grafting of SCNPs into the main chain prevents nanoparticle aggregation in solution, unlike in traditional NP/polymer melt composite systems, due to the bonding effect. The viscosity curves (Fig. 5f) demonstrate that increasing T-SCNP content results in a progressive decrease in system viscosity, indicating the easier occurrence of chain entanglements. The minimum viscosity ratio (*η*/*η*_pure_) reaches 0.08 at a 10% grafting content, reflecting a viscosity reduction of over 90%. Moreover, the *τ*_R_/*τ*_R pure_ ratio decreases to 0.13 as the grafted content of the T-SCNPs increases (Fig. 5g). Thus, the rheological behavior exhibited by the comb of T-SCNPs in solution is intricately linked not only to the size of the SCNPs but also to their grafting density.

## Conclusions

In this study, the rheological properties of a specialized superstructure polymer, referred to as the comb of T-SCNPs, were examined. This polymer utilizes tadpole-type SCNPs (T-SCNPs) with active end chains as reaction units. A comb of T-SCNPs was synthesized, with a focus on parameters such as the relaxation time of the chains (*τ*_R_) and mesh size (*ξ*). The microstructure of these polymers is critical in determining their macroscopic properties. Compared with traditional SCNP and linear matrix resin composites (SCNPs&H-LP) as well as traditional comb polymers (comb of F-LPs), the comb of T-SCNPs results in a smaller *ξ* and faster *τ*_R_. These characteristics result in fewer entanglements among chains within the comb of the T-SCNP system, leading to reduced viscosity and loss modulus. The introduction of SCNPs into the system through grafting effectively decreases the local density of chains, thereby accelerating the disentanglement time with increasing frequency. Furthermore, the findings establish a direct correlation between the rheological behavior of the comb of T-SCNPs and the size and content of grafted SCNPs, suggesting that larger sizes or higher contents of grafted SCNPs result in lighter chain entanglement, quicker chain relaxation time, larger system mesh size, and decreased loss modulus and viscosity.

## Author contributions

Hongting Pu planned and designed the project. Yangjing Chen conducted all of the experiments. Zhiyu Hu, Zhigang Shen, and Xiaoqiang Xue helped synthesize the materials. Zhiyu Hu helped characterize the samples. Yangjing Chen and Hongting Pu analyzed the data and wrote the paper. All authors reviewed the manuscript.

## Conflicts of interest

There are no conflicts to declare.

## Supplementary Material

SC-015-D4SC05650G-s001

## Data Availability

The data supporting this article have been included as part of the ESI.†
