# Supplementary material for: Preparation of superstructured comb polymers based on tadpole-shaped single-chain nanoparticles
Source: Chem Sci. 2024 Oct 3;15(42):17590–9. doi: 10.1039/d4sc05650g (PMC11457303; doi:10.1039/d4sc05650g)
Supplement: SC-015-D4SC05650G-s001 [file SC-015-D4SC05650G-s001.pdf]

Supplementary Information (SI) for Chemical Science.
This journal is © The Royal Society of Chemistry 2024

Electronic Supplementary Information

Preparation of Superstructured Comb Polymer Based on Tadpole-Shaped Single-Chain Nanoparticles

Yangjing Chen[1], Zhiyu Hu[1], Zhigang Shen[2], Xiaoqiang Xue[3], and Hongting Pu*[1]

[1]Department of Polymer Materials, School of Materials Science & Engineering, Tongji University, Shanghai, 201804, China

[2]Sinopec Shanghai Research Institute of Petrochemical Technology Co., LTD., Shanghai, 201208, China

[3]Industrial College of Carbon Fiber and New Materials, School of Chemical Engineering and Materials, Changzhou Institute of Technology, Changzhou, Jiangsu 213000, China

SUPPORTING INFORMATION

Table of Contents

1. Chemicals and materials 2

2. Instrumentations and measurements...2

3. Synthetic procedures..3-5

4. Supporting Figures..6-29

5. Table S1-3..30

6. References 31

Experimental Procedures

1. Chemicals and materials

Methyl methacrylate (MMA, 99 %) was distilled before use, anthracenyl methacrylate (AMA), *α*-bromoisobutyryl bromide, Hydrazine hydrate (80 %), triethyl amine (TEA), *N, N, N′ N″, N″*-pentamethyldiethylenetriamine (PMDETA), tetrahydrofuran (THF), dichloromethane (CH_2Cl_2) and methanol were supplied by Aladdin Reagent Co. (China). tert-butyl 2-bromoisobutyrate (*t*-BBiB, 99 %, Shanghai Reagent Factory, China), 4-((4-hydroxyphenyl)diazenyl) benzaldehyde (Azo-CHO) and methacrylate hydrazide ester (MAH) were prepared according to the previous literature.[1,2] 2,2'-Azobisisoheptonitrile (AIBN, 98%) was recrystallized with ethanol before use; CuBr (96 %, Shanghai Reagent Factory, China) was purified by stirring with glacial acetic acid followed by filtering and washing the resulting solid with ethanol). All other reagents were of analytical grade, purchased from Shanghai Chemical Reagent Co.(China), and used as received.

2. Instruments and methods

TD-SEC. The molecular weights and polydispersity of polymers were measured at 35 °C by SEC in THF using a Waters 1515 instrument equipped with a Waters 2414 differential refractive index (dRI) detector, a Wyatt DAWN HELEOS-II multi-angle laser-light scattering (MALLS) detector, and a Wyatt Visco Star viscometer detector (VD). HPLC-grade THF at a flow rate of 1.0 mL/min was used as the eluent. A series of narrowly dispersed polystyrene (PS) standards were used for calibrating the number-average molecular weight ($M_{n\ SEC}$) and the molecular weight distribution (*Đ*).

^{1}H-NMR. Monomer and polymer structures were determined using a Bruker ARX-500 type NMR spectrometer at 25 °C with deuterated chloroform ($CDCl_3$) and dimethyl sulfoxide (DMSO) as the solvents and tetramethylsilane (TMS) as the internal standard. The hydrodynamic radius (R_h) was acquired by dynamic light scattering (DLS) using an ALV/CGS-3 compact goniometer system at 25°C, and a He-Ne laser operating at a wavelength of λ_0 = 632.8 nm was used as a light source. Sample solutions for analysis (2 mg/mL) were poured into the sample bottle, which was placed in a sample cell filled with toluene used as the immersion liquid. Temperature control of the sample was provided by an external

thermostated circulating bath.

UV spectra. UV spectra were recorded on Hitachi U-2900. The photodimerization degree (PD) was figured out from UV-Vis spectra by comparing the peak absorption at 365 nm assigned to the anthracene group.

Diffusing wave spectroscopy. The microrheological behaviors of polymers and polymer networks were determined by Diffusing Wave Spectroscopy (DWS, Swiss LS instrument company), and titanium dioxide (TiO_2) with a particle size of 360 nm were used as tracer particles. The samples dissolved in THF for DWS measurement were well mixed with 0.5 wt% particles, and an ultrasonicator was also used to ensure the homogeneity of the samples by ultrasonic mixing. Scattering from the sample was measured in a 1 mm glass cell on an LS Instruments RheoLab system. The transport means free path *l** was determined from the control sample with the same size particles in THF.

Differential scanning calorimetry. Thermal analysis of the polymers was performed by differential scanning calorimetry (DSC) using a TA instrument DSC 2010 with a heating and cooling rate of 10 °C/min under a continuous nitrogen flow.

Small-angle X-ray scattering. SAXS was carried out at the beamline BL16B of the Shanghaisynchrotron radiation facility (SSRF, China). The energy of the incident X-rays used for the SAXS experiments was 18 KeV. To measure the aggregate structures, the measurement scale range during the SAXS experiments was adjusted to cover 1-10 nm. The exposure time was 60 s for each test. The scattered X-ray intensities were recorded using the 2-D plate imaging technology. The absorption of X-rays by the sample and the background scattering were corrected. All the absorption coefficients of the samples were normalized by the data recorded from the ionization chambers. The measurements were performed at room temperature on solutions of polymers in DMF at a concentration of 5 mg/mL in capillaries of 2 mm thickness.

Synthetic procedures

1. Synthesis of ATRP initiator containing aldehyde groups (CHO-Br)

As shown in Scheme S1, a 250 mL round bottom flask was added 4-((4-hydroxyphenyl)diazenyl) benzaldehyde (11.5 g, 0.05 mol), TEA (6.06 g, 0.06 mol) and CH_2Cl_2 (50 mL), then the solution was

stirred in ice bath. α-bromoisobutyryl bromide (7.38 mL, 0.06 mol) diluted in dry CH_2Cl_2 (30 mL) was then added to the cool stirred mixture dropwise and the resultant mixture was vigorously stirred for additional 5 h at 0-5°C after addition. The mixture was filtered, then filtrate was dissolved in hydrochloric acid solution, washed with 5% Na_2CO_3 aqueous solution, and deionized water three times. The organic phase was dried with anhydrous $MgSO_4$. After the removal of solvent under reduced pressure, the colorless product was obtained (Yield: 13.5 g, 53.1%). The purity is above 98.3% (Fig. S1). ^{1}H-NMR (500 MHz, DMSO, Fig. S2), δ (TMS, ppm): 10.2 ppm (a, 1H, -CHO-), 7.3-7.5 ppm (b, Ar), 7.9-8.1 ppm (c, Ar), 1.9-2.0 ppm (e, 6H, $-(CH_3)_2$).

2. Synthesis of macro-initiator CHO-PMMA-Br

For the synthesis of CHO-PMMA-Br: MMA (3.00 g, 30 mmol), CHO-Br (378.0 mg; 1 mmol), PMDETA (173 mg; 1 mmol) and toluene (3 mL) were added into a 50 mL Schlenk tube. The solution was degassed by three freeze-pump-thaw cycles. In the last cycle, CuBr (143 mg; 1.00 mmol) was added to the frozen solution under N_2 flow, after which the frozen solution was evacuated again. The tube was then back-filled with N_2 and the frozen solution was melted, and placed in an oil bath at 70 °C. The polymerization was terminated after 8 h by cooling the reaction mixture, and the mixture was diluted with THF, passed through an alumina column to remove Cu complexes, and precipitated in methanol. Finally, drying in a vacuum at 40 °C for 24 h. (Yield (%) = 86.2, $M_{n\ SEC} = 3.2 \times 10^3$ g/mol, $Đ = 1.18$).

3. General procedure for addition of co-monomers (MMA and AMA) to polymer chain ends via ATRP

Typical synthesis procedure for *F*-LP: MMA (4.5 g; 45 mmol), AMA (1.31 g; 5 mmol), CHO-PMMA-Br (1.8 g; 0.5 mmol), PMDETA (86.5 mg; 0.5 mmol), toluene (5 mL) and anisole (1 mL) were added into a 20 mL Schlenk tube. The solution was degassed by three freeze-pump-thaw cycles. In the last cycle, CuBr (71.5 mg; 0.5 mmol) was added to the frozen solution under N_2 flow, after which the frozen solution was evacuated again. The tube was then back-filled with N_2 and the frozen solution was melted. Then the tube was sealed and placed in an oil bath at 70 °C. The polymerization was conducted for 24 h and was terminated by cooling the flask. The mixture was diluted with THF and passed through

an alumina column to remove Cu complexes, and precipitated in methanol. Finally, drying in a vacuum at 40 °C for 24 h, yielded PMMA-*b*-P(MMA-*r*-AMA) (*F*-LP). (Yield (%) = 79.2, $M_{n\,SEC}$ = 1.5 ×10^4 g/mol, $Đ$ = 1.52, R_h = 6.4 nm).

4. Synthesis of tadpole single-chain nanoparticles (*T*-SCNP) via intramolecular photodimerization of anthracene units

F-LP (0.1 g) was dissolved in dry THF (200 mL) and stirred at room temperature for 2 h under nitrogen atmosphere in dark. It was then illuminated in a photo-reactor under magnetic stirring for 2 h. At the end of the period, solution was concentrated by evaporation and polymer was precipitated in excess methanol. The final polymer was filtered and dried in a vacuum oven at 40 °C. (*T*-SCNP: Yield (%) = 69.4, $M_{n\,SEC}$ = 1.3×10^4 g/mol, $Đ$ = 1.31, R_h = 5.2 nm).

5. Synthesis of high molecular weight linear carrier polymer (*H*-LP)

For the synthesis of *H*-LP, MMA (8.0 g, 80 mmol), MAH (2.0 g, 20 mmol), and AIBN (16.4 mg, 0.1 mmol) were dissolved in DMSO (10.0 mL). The reaction mixture was degassed by passing argon for 30 min. The copolymerization reaction was carried out at 65 ºC for 18 h. The polymerization reaction was quenched after a certain reaction time by cooling to room temperature. Finally, the polymer was precipitated from the solution by the addition of a large excess of methanol. The precipitate was isolated by filtration and dried at room temperature under vacuum. (*H*-LP: Yield (%) = 95.2, $M_{n\,SEC}$ = 5.6×10^4 g/mol, $M_{n\,SEC}$ = 10.8×10^4 g/mol, $Đ$ = 1.82, composition (^{1}H-NMR) = 21.2 mol% MAH).

6. Preparation of comb polymer based on *T*-SCNPs (comb of *T*-SCNPs)

T-SCNPs (0.3 g) and *H*-LP (0.1g) were dissolved in 4 mL of DMF and then stirred at room temperature for 24 h. The resultant polymer was precipitated from the solution by the addition of a large excess of methanol. The precipitate was isolated by filtration and dried at room temperature under vacuum.

Results and Discussion

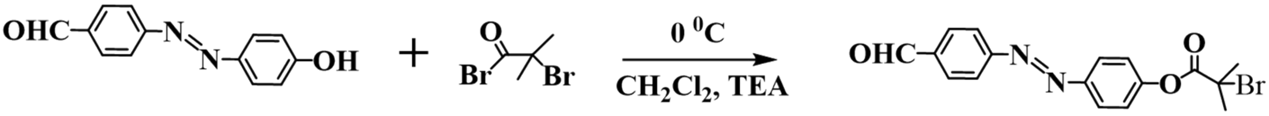

Scheme S1. The synthesis route of ATRP initiator (CHO-Br)

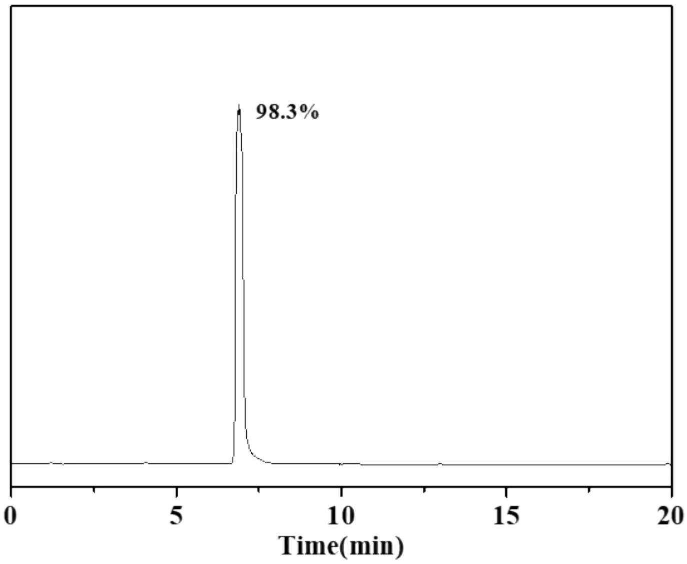

Fig. S1 HPLC result of ATRP initiator (CHO-Br).

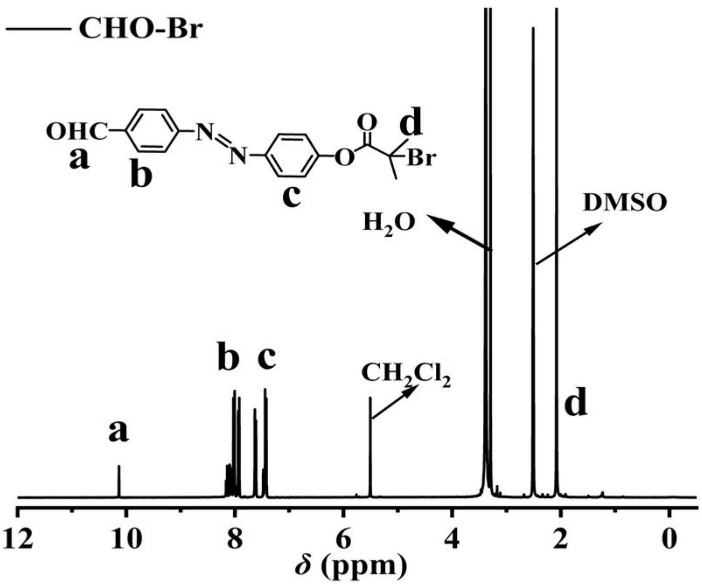

Fig. S2 ^{1}H-NMR of ATRP initiator (CHO-Br) in DMSO.

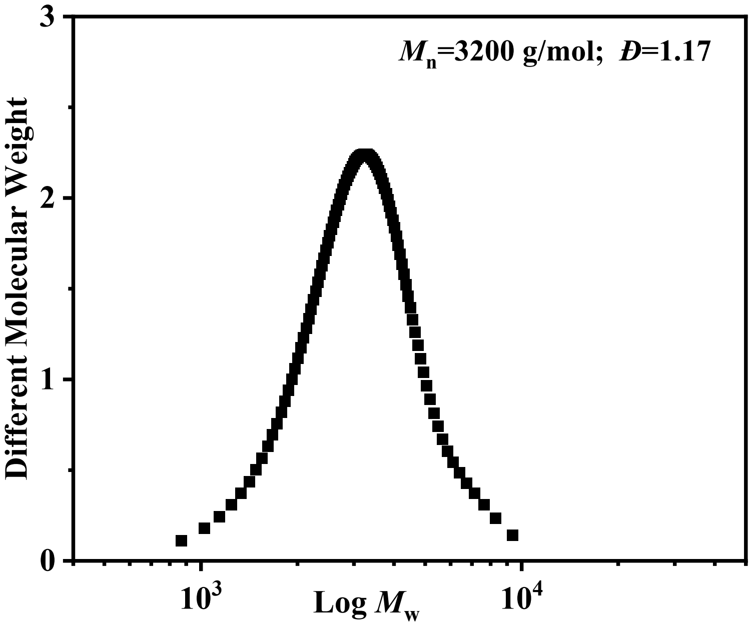

Fig. S3 SEC curve of macroinitiator CHO-PMMA-Br.

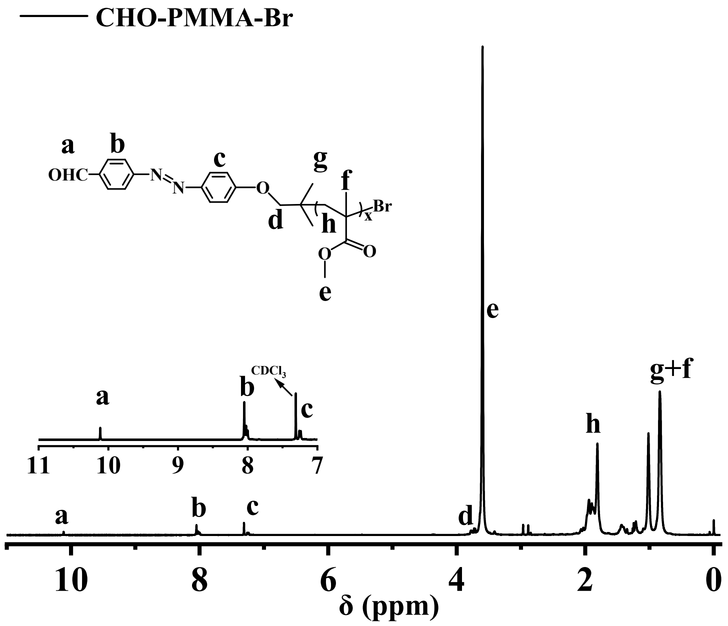

Fig. S4 ^{1}H-NMR of the macroinitiator CHO-PMMA-Br in $CDCl_3$.

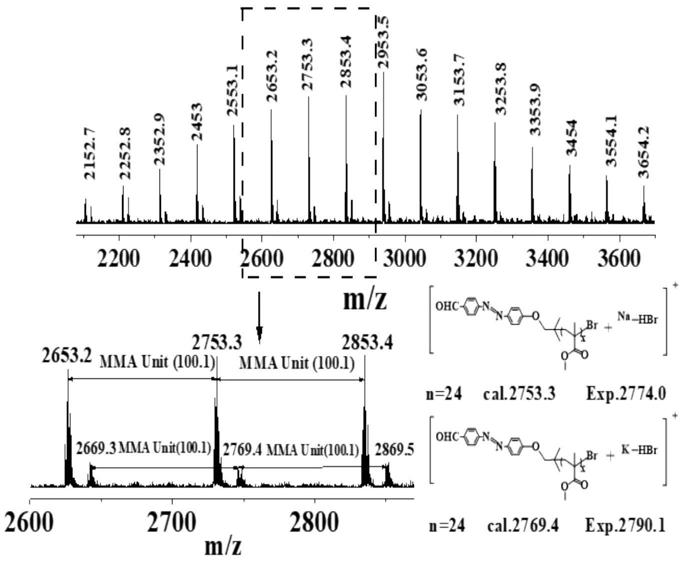

Fig. S5 The Mass spectrometry (MALDI-TOF-MS) of the macroinitiator CHO-PMMA-Br.

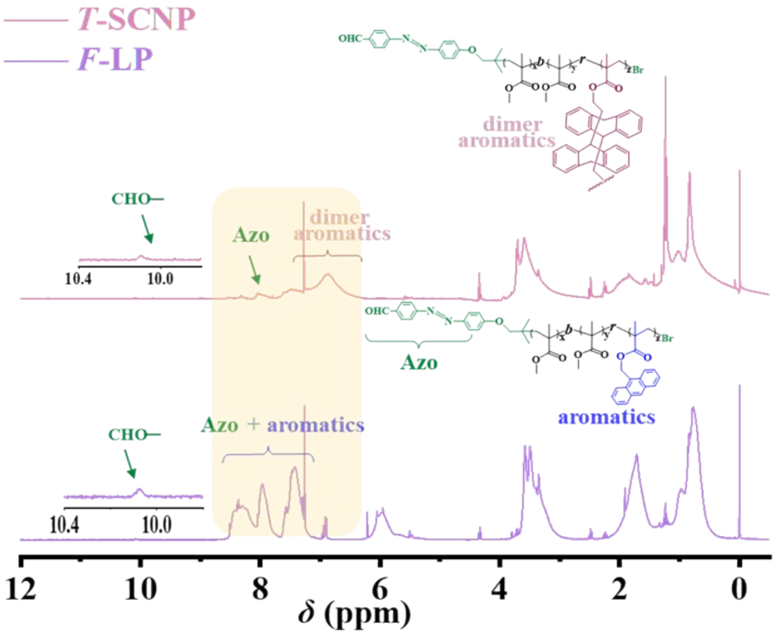

Fig. S6 ^{1}H-NMR spectra of *F*-LP and *T*-SCNP

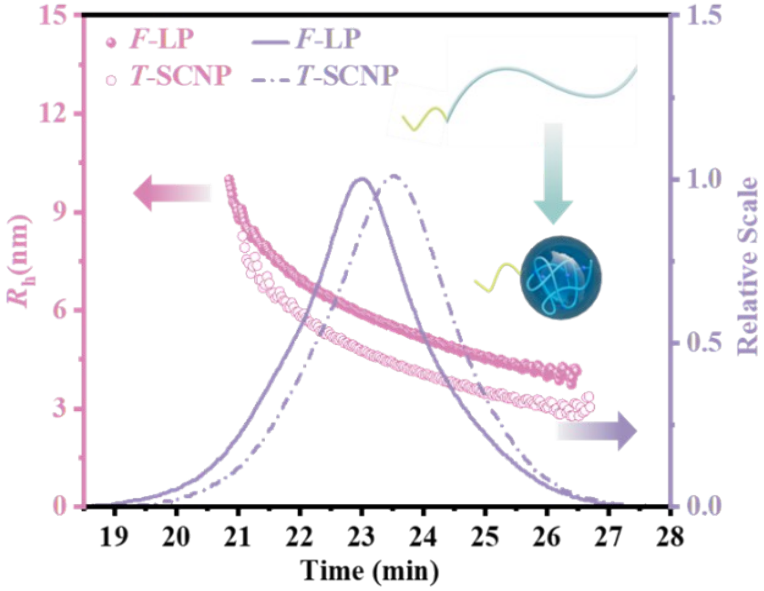

Fig. S7 SEC profiles and hydrodynamic radius (R_h) of *F*-LP and *T*-SCNP

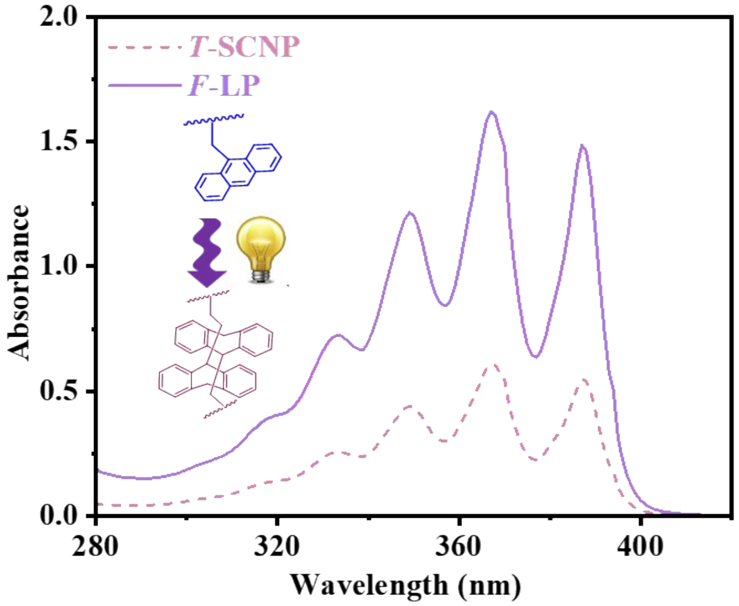

Fig. S8 UV-Vis absorption spectra of *F*-LP and *T*-SCNP

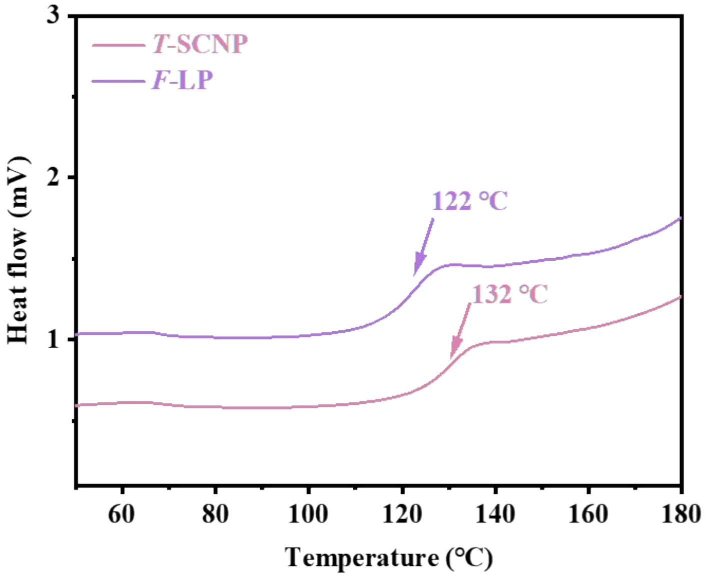

Fig. S9 DSC profiles of *F*-LP and *T*-SCNP

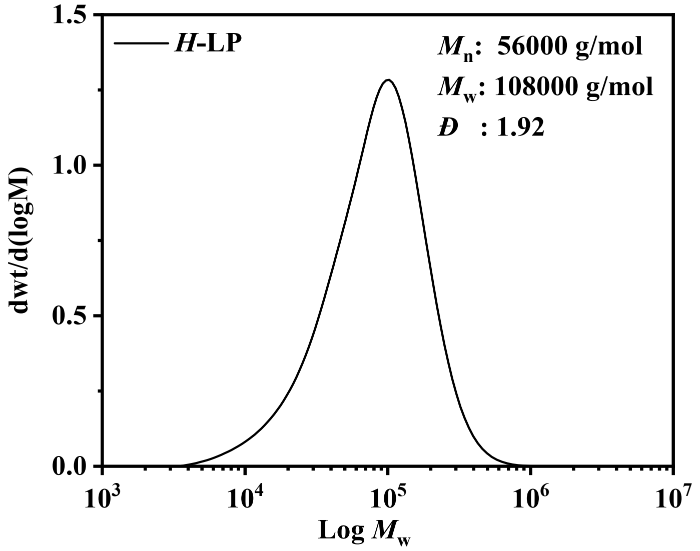

Fig. S10 SEC curve of high molecular weight linear polymer (*H*-LP).

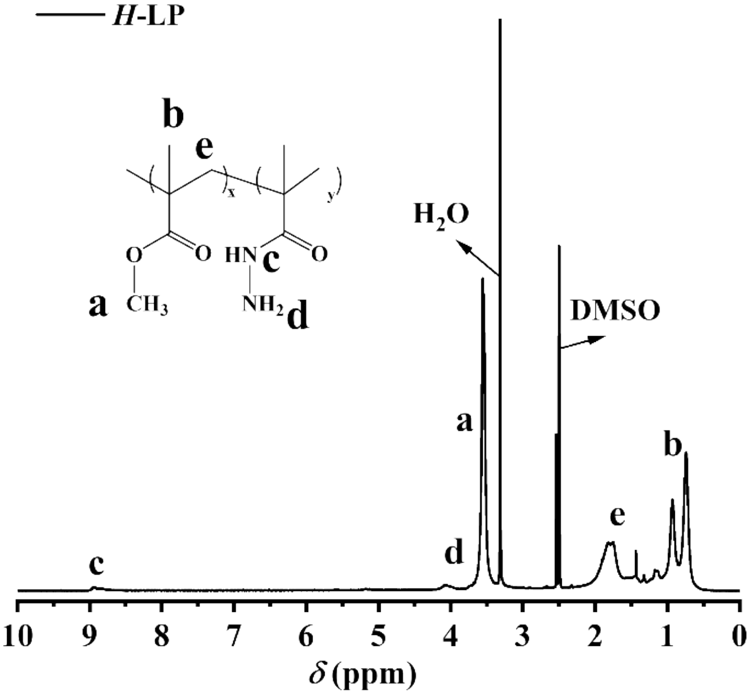

Fig. S11 ^{1}H-NMR of *H*-LP in DMSO.

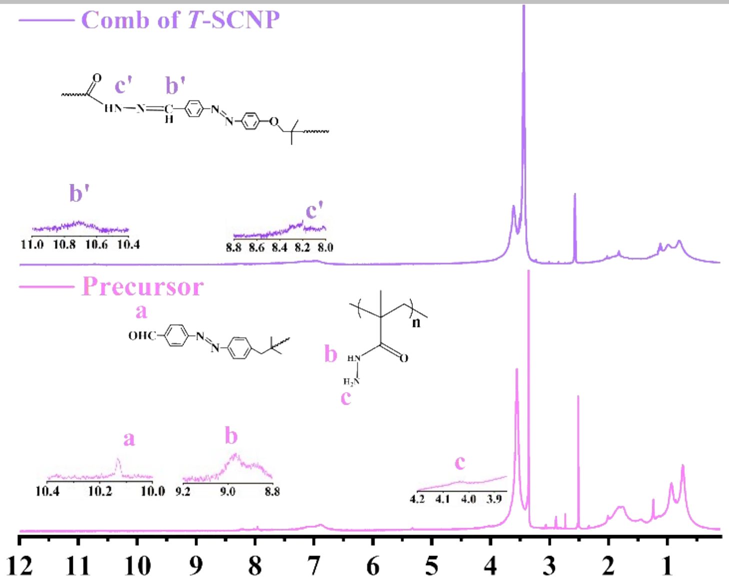

Fig. S12 ^{1}H-NMR spectra of comb of *T*-SCNPs and its precursor.

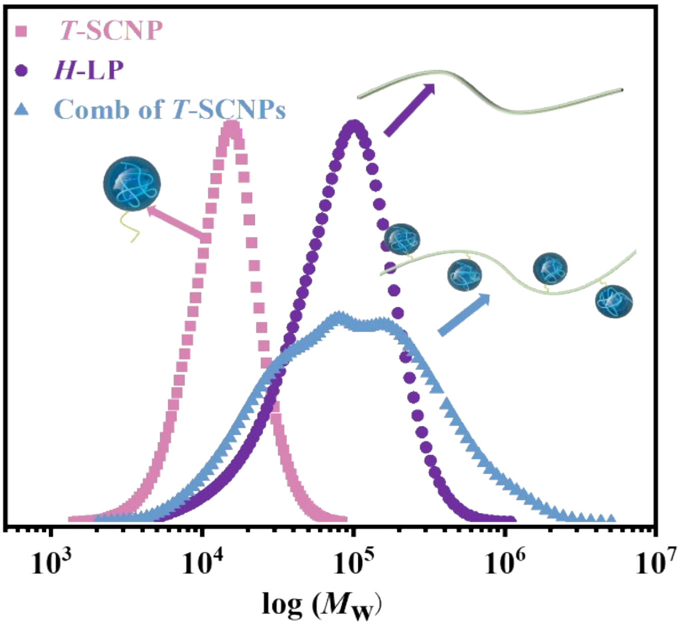

Fig. S13 SEC curves of *T*-SCNPs and its precursor.

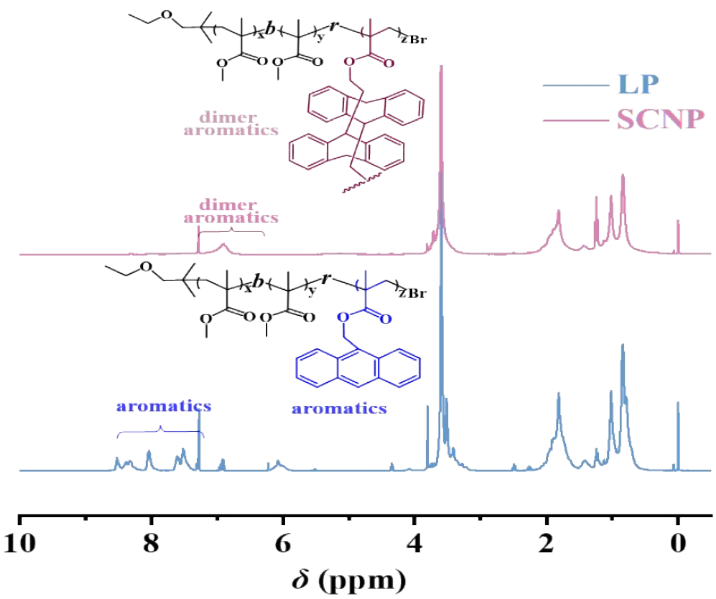

Fig. S14 ^{1}H-NMR result of LP and SCNP in $CDCl_3$.

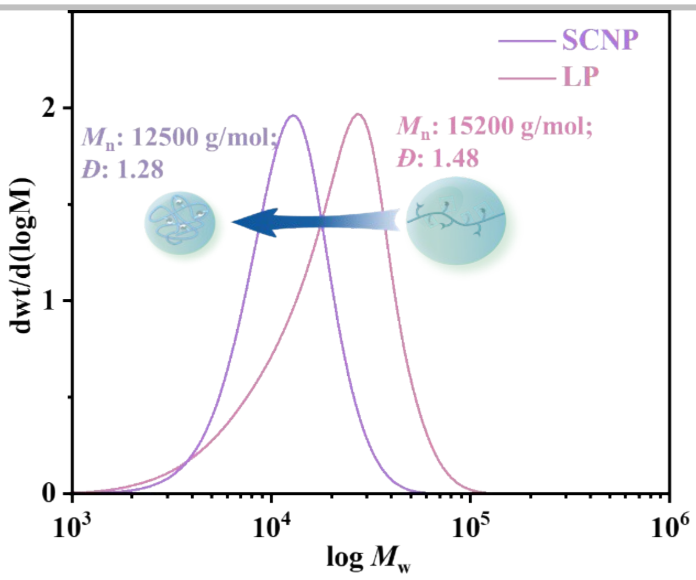

Fig. S15 SEC curves of LP and SCNP.

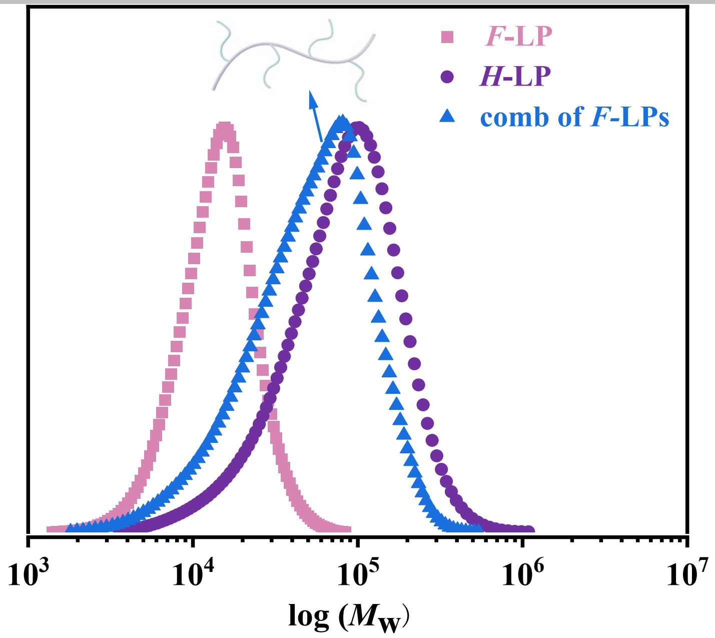

Fig. S16 SEC curves of *F*-LP, *H*-LP and comb of *F*-LPs.

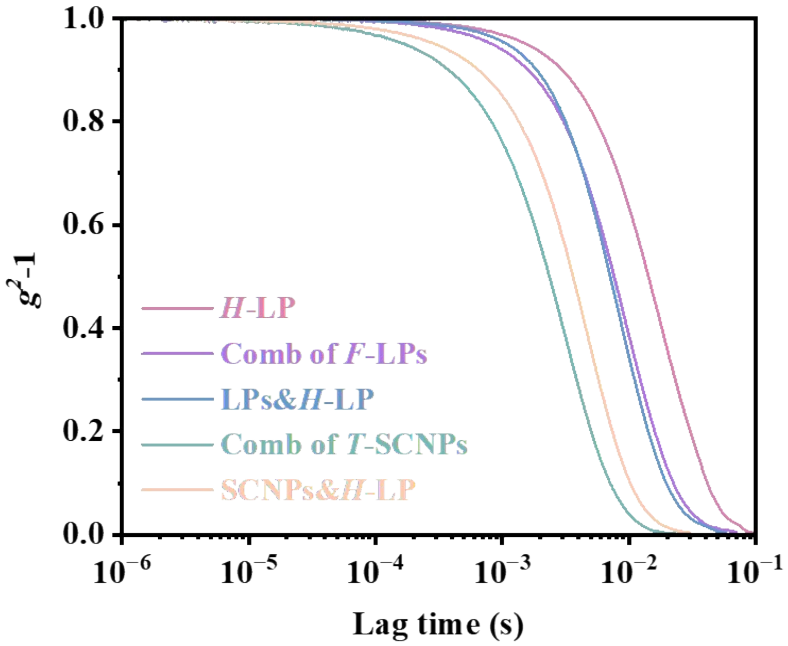

Fig. S17 ICF curves of different systems.

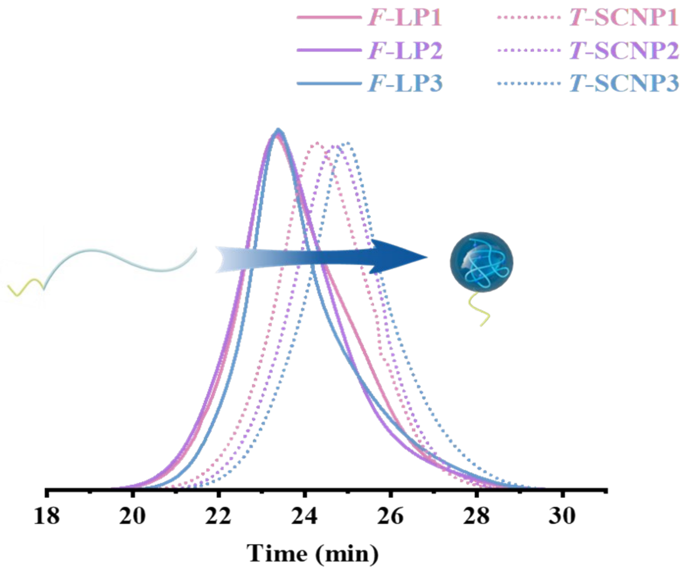

Fig. S18 SEC curves of *T*-SCNPs with different sizes and their linear precursors.

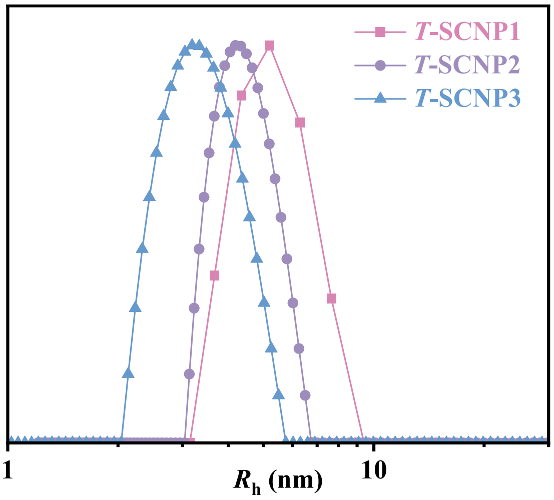

Fig. S19 Hydrodynamic radius (R_h) of *T*-SCNPs with different sizes.

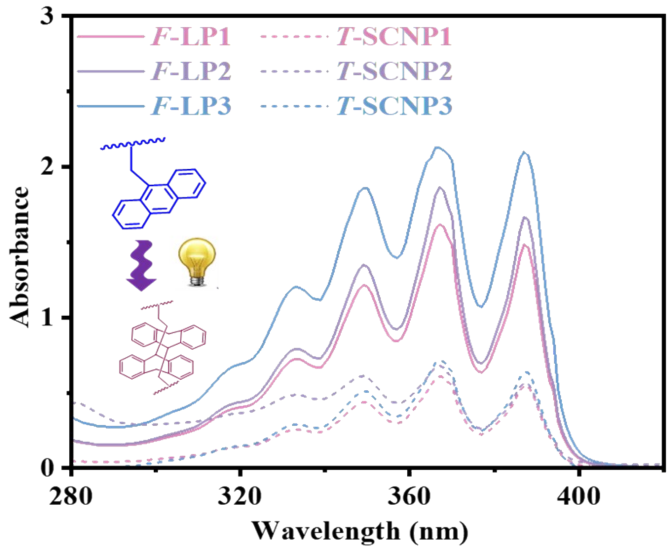

Fig. S20 UV-Vis absorption spectra of *T*-SCNPs with different sizes and their linear precursors.

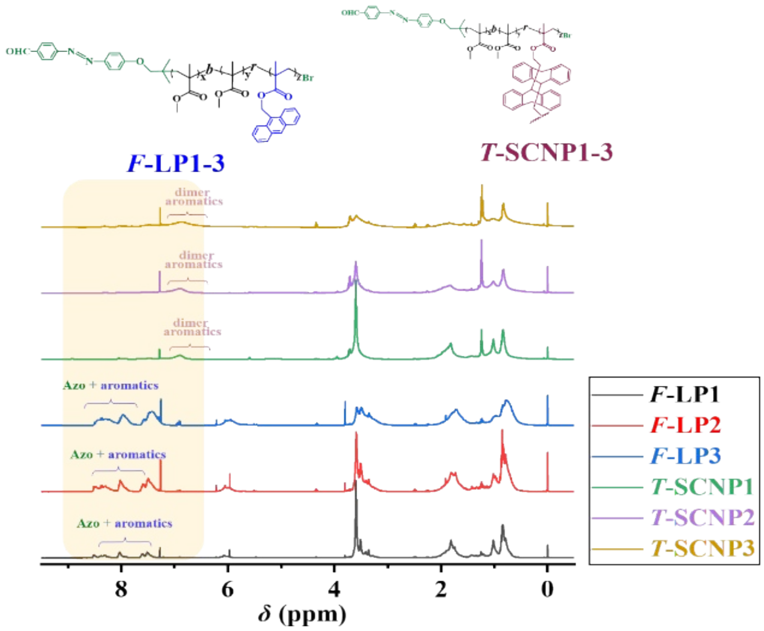

Fig. S21 ^{1}H-NMR result of *F*-LP1-3 and corresponding *T*-SCNPs.

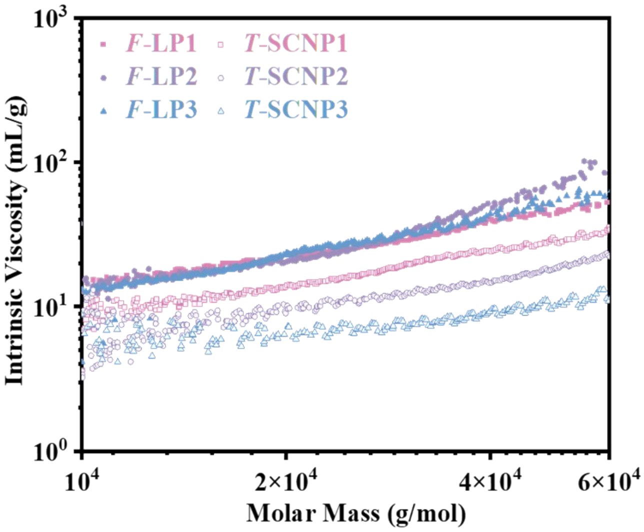

Fig. S22 The intrinsic viscosity of *T*-SCNPs with different sizes and their linear precursors.

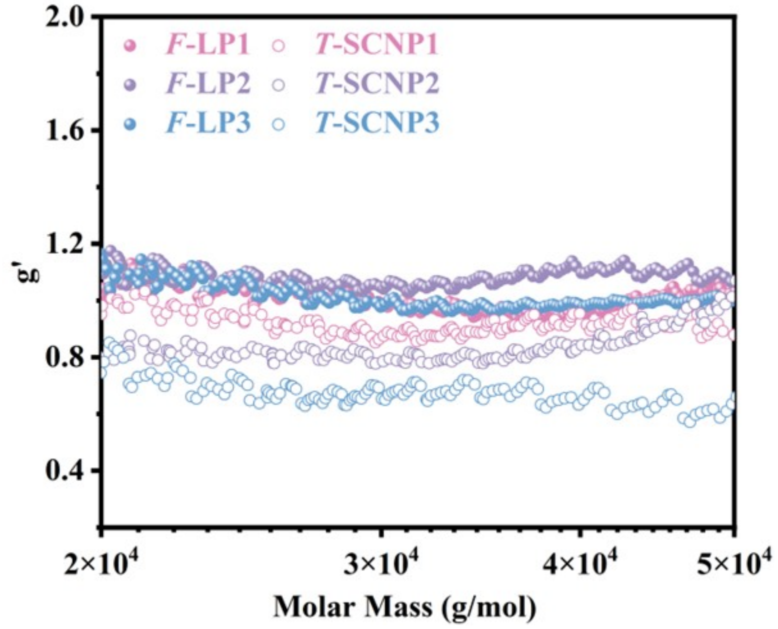

Fig. S23 *g′* values of precursor polymers (*F*-LPs) and single-chain nanoparticles (*T*-SCNPs) with different size

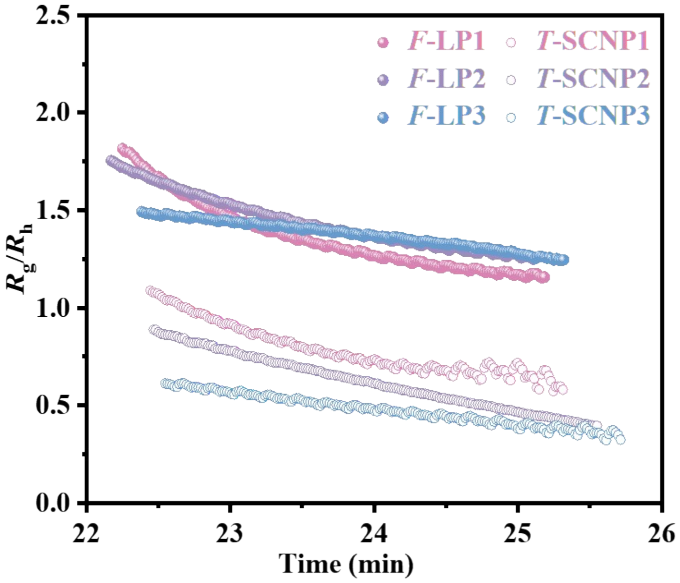

Fig. S24 The R_g/R_h curves of *T*-SCNPs with different sizes and their linear precursors.

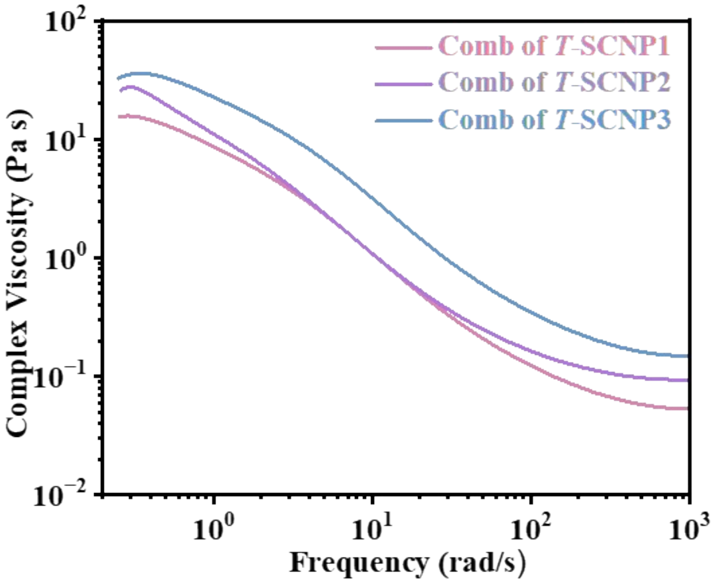

Fig. S25 Complex viscosity curves of comb of *T*-SCNPs with different sizes of *T*-SCNPs.

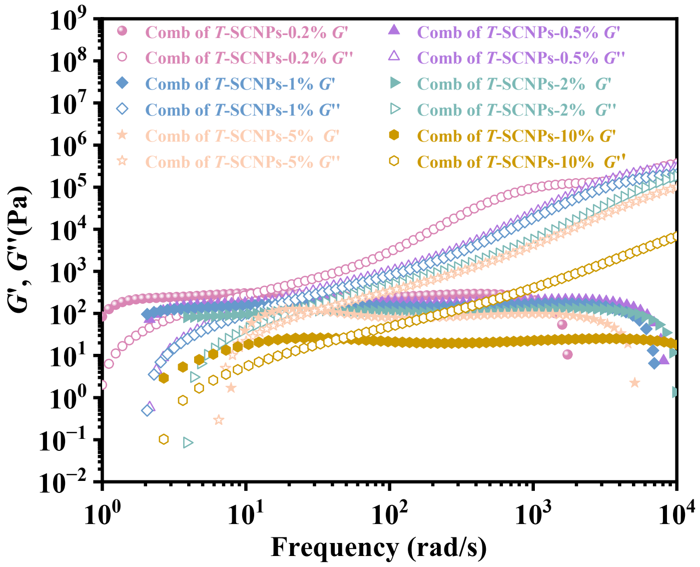

Fig. S26 Frequency spectra of G' and G'' of comb of T-SCNPs with diffent contents of T-SCNPs.

Table S1. Characteristics of precursor polymer (*F*-LP) and single-chain nanoparticles (*T*-SCNP)

Samples	$M_{n\ SEC}$ [a] (g/mol)	$M_{w\ MALLs}$ [a] (g/mol)	$Đ$[a]	R_h [b]	T_g (°C)
F-LP	15300	21400	1.52	6.4	122
T-SCNP	13200	19100	1.31	5.2	133

a Determined by SEC
b Hydrodynamic diameter (R_h) was measured by DLS in toluene; the concentration of the samples was 2 mg·mL^{-1}.

Table S2. Characteristics of precursor polymers (*F*-LPs) and single-chain nanoparticles (*T*-SCNPs) with different size

Samples	$M_{n\ SEC}$ (g/mol)	$Đ$	R_h (nm)	Intrinsic viscosity (pa.s)
F-LP1	15300	1.52	6.4	17.99
T-SCNP1	13200	1.31	5.2	14.95
F-LP2	15700	1.51	6.6	18.35
T-SCNP2	12300	1.28	4.4	13.09
F-LP3	16100	1.52	6.1	17.76
F-SCNP3	10300	1.36	3.5	7.98

Table S3. Microrheological properties of comb of *T*-SCNPs with different grafting contents

Samples	$6\delta^2\times10^5$ [a] (um^2)	η_0 [a] (Pa s)	$\eta_m\times10^2$ [a] (Pa s)	G_0 [a] (Pa)	$\tau_R\times10^2$ [b] (s)	Mesh size [c] (nm)
Comb of *T*-SCNP-0.2%	6.92	89.6	21.2	243	10.1	25.54
Comb of *T*-SCNP-0.5%	7.82	70.8	16.9	182	8.2	28.12
Comb of *T*-SCNP-1%	9.73	52.5	12.2	138	6.3	30.85
Comb of *T*-SCNP-2%	13.28	33.3	6.2	111	5.3	33.17
Comb of *T*-SCNP-5%	16.32	17.9	5.8	88	4.3	35.83
Comb of *T*-SCNP-10%	61.42	9.8	2.6	21	2.64	57.82

(1) Xue, X. Q.; Liang, K.; Huang, W. Y.; Yang, H. J.; Jiang, L.; Jiang, Q. M.; Jiang, T.; Lin, B. Z.; Chen, Y. J.; Jiang, B. B.; Komarneni, S. Molecular Engineering of Injectable, Fast Self-Repairing Hydrogels with Tunable Gelation Time: Characterization by Diffusing Wave Spectroscopy. *Macromolecules* **2022**, *55* (15), 6474-6486.
(2) Ballard, N.; Bon, S. A. F. Dynamic uptake and release from poly (methacryloyl hydrazide) microgel particles through reversible hydrazide-aldehyde chemistry. *Polym. Chem.* **2014**, *5* (23), 6789-6796.
